# Evaluation of Humoral Response following SARS-CoV-2 mRNA-Based Vaccination in Liver Transplant Recipients Receiving Tailored Immunosuppressive Therapy

**DOI:** 10.3390/jcm12216913

**Published:** 2023-11-03

**Authors:** Tommaso Maria Manzia, Bruno Sensi, Luigi Eduardo Conte, Leandro Siragusa, Roberta Angelico, Francesco Frongillo, Giuseppe Tisone

**Affiliations:** 1 Department of Surgical Science, Università degli Studi di Roma “Tor Vergata”, 00133 Rome, Italybrunosensi@outlook.it (B.S.);; 2 Department of Surgery-Transplantation Service, Catholic University of the Sacred Heart, 00168 Rome, Italy

**Keywords:** immunosuppression, tailored therapy, liver transplantation, SARS-CoV-2, COVID-19, vaccines

## Abstract

**Background**: The role of tailored immunosuppression (IS) in the development of the humoral response (HR) to SARS-CoV-2 mRNA-based vaccination in liver transplant (LT) recipients is unknown. **Methods**: This is a single-centre prospective study of patients who underwent LT between January 2015 and December 2021 and who have received three doses of mRNA-based SARS-CoV-2 vaccination. Patients undergoing Tacrolimus-based immunosuppression (TAC-IS) were compared with those undergoing Everolimus-based immunosuppression (EVR-IS). Patients receiving the TAC-EVR combination were divided into two groups based on trough TAC concentrations, i.e., above or below 5 ng/mL. HR (analysed with ECLIA) was assessed at 30 to 135 days after vaccination. The primary outcome was the presence of a positive antibody titre (≥0.8 U/mL). Secondary outcomes were the presence of a highly protective antibody titre (≥142 U/mL), median antibody titre, and incidence of COVID-19. **Results**: Sixty-one participants were included. Twenty-four (40%) were receiving TAC-IS and thirty-seven (60%) were receiving EVR-IS. At the median follow-up of 116 (range: 89–154) days, there were no significant differences in positive antibody titre (95.8% vs. 94.6%; *p* = 0.8269), highly-protective antibody titre (83.3% vs. 81.1%; *p* = 0.8231), median antibody titre (2410 [IQ range 350–2500] vs. 1670 [IQ range 380–2500]; *p* = 0.9450), and COVID-19 incidence (0% vs. 5.4%; *p* = 0.5148). High serum creatinine and low estimated glomerular filtration rate were risk factors for a weak or absent HR. **Conclusions**: Three doses of mRNA-based SARS-CoV-2 vaccination yielded a highly protective HR in LT recipients. The use of TAC or EVR-based IS does not appear to influence HR or antibody titre, while renal disease is a risk factor for a weak or null HR.

## 1. Introduction

The SARS-CoV-2 pandemic had a large impact on most of the world’s population, but it primarily and more deeply affected vulnerable patients with poor prognostic factors. Liver transplantation (LT) is the only effective life-saving treatment for end-stage liver disease and malignant hepatic neoplasms. However, recipients require life-long immune-suppression (IS), which places them in the “fragile” category. There are several effective IS regimens and, today, they are usually tailored to the individual patient [1,2]. In general, tacrolimus (TAC) has been the historical cornerstone of most therapies and has recently been found to be “protective” in COVID-19 disease compared to other IS regimens [3,4]. This is an important finding as COVID-19 has high rates of hospitalisation, intensive care admission, and mortality in non-vaccinated LT patients [5,6,7,8]. SARS-CoV-2 vaccination may have a large impact on the development of disease. For this reason, it is important to investigate whether certain IS regimens influence the immune response to vaccination. Mycophenolate seems to be particularly harmful in this regard, while some studies have reported benefits of maintaining low TAC levels [9,10]. As TAC is the most commonly used IS regimen in LT patients, we sought to further investigate the role of TAC-based IS (TAC-IS) on the immune response to SARS-CoV-2 vaccination. Humoral response (HR) can be easily measured with common laboratory tests and is therefore the most commonly used surrogate for immune response. However, it does not give a measure of other aspects of the immune response, such as cellular immunity. The aim of this study was to compare the HR to SARS-CoV-2 mRNA vaccines in LT patients taking TAC-IS and those taking Everolimus (EVR-IS).

## 2. Materials and Methods

Eligible patients were identified from a prospectively maintained database and prospectively enrolled in the HR assessment. The study was conducted according to the international ethical recommendations on clinical research established by the Helsinki Declaration and in accordance with the STROBE criteria (https://strobe-statement.org (accessed on 10 April 2023)) [11]. The study was approved by the centre’s independent ethical committee and registered at ClinicalTrial.Gov (Identifier: NCT05490342).

### 2.1. Patients

All patients with available follow up who underwent LT at AOU Policlinico Tor Vergata between 1 January 2015 and 31 December 2021 were evaluated for the study. Inclusion criteria were as follows: (i) patients older than eighteen years of age who underwent LT for any underlying aetiology and had received three doses of SARS-CoV-2 vaccination with BNT162b2 (Pfizer-BioNTech) or mRNA-1273 (Moderna-NIAID) SARS-CoV-2 and (ii) patients who received de novo EVR and TAC after LT, including those who were subject to IS minimisation during their follow up in accordance with the Italian Consensus Guidelines [12]. Additionally, exclusion criteria were as follows: (i) patients diagnosed with COVID-19 before vaccination; (ii) patients who did not receive vaccination; (iii) patients who had received fewer than or more than three doses; and (iv) patients under mycophenolate.

### 2.2. Study Design

This is a prospective single-centre study comparing the HR to SARS-CoV-2 vaccination of LT recipients taking different IS regimens. Patients who were taking TAC-IS at the time of vaccination were compared with patients taking EVR-based IS. TAC-IS was defined as either TAC monotherapy or a TAC serum trough concentration above 5 ng/mL when also taking Everolimus (EVR) (Certican, Novartis Pharma, Basel, Switzerland). EVR-IS was defined as either TAC-free therapy or as combined therapy with a TAC serum trough concentration below 5 ng/mL [3,13]. The TAC serum trough concentration used to group the patients was measured during the vaccination cycle. If patients did not have such measurements, the most recent TAC concentration was used. If they had multiple TAC level tests during the vaccination cycle, the average concentration was used. The study involved no modification of IS management by the transplant team.

### 2.3. HR Assessment Methodology

HR testing was performed between 30 and 135 days after administration of the third vaccine dose. In all patients, HR was evaluated by electrochemiluminescence immunoassay (ECLIA), which determines the level of in vitro antibodies, including IgG, to the SARS-CoV-2 spike protein (S) anti-RBD (receptor-binding domain) in serum and plasma samples. Specifically, the tool used was Elecsys^®^ SARS-CoV-2 S, Roche, Basel, Switzerland. The results were obtained either in U/mL or binding antibody units (BAU)/mL and considered to be comparable regardless of the unit of measurement used (without need for conversion) as per the World Health Organisation (WHO) international standard for anti-SARS-CoV-2 immunoglobulin (NIBSC code 20/136) [14].

### 2.4. Outcome Measures

The primary outcome was the presence of a positive antibody titre, defined as a titre ≥ 0.8 U/mL. Secondary outcomes were the presence of highly protective antibody titres; the median antibody titres; and the subsequent development of COVID-19. A highly protective antibody titre was defined as a titre ≥ 142 U/mL. Predictive factors for a highly protective antibody response were also investigated.

### 2.5. Study Variables

Pre-vaccination data included the following factors: age, gender, IS regimen, LT data, BMI, comorbidities, previous SARS-CoV-2 infection, and laboratory tests. Post-vaccination data collected included the type of vaccine administered; the vaccine–ECLIA interval; and whether the patient subsequently contracted COVID-19.

### 2.6. Statistical Analysis

A non-parametric approach was preferred in the statistical analysis due to the limited sample size. Continuous variables were described using the median and first and third quartiles. Categorical variables were described using absolute frequencies and percentages. Group comparisons were made using Fisher‘s exact test and the Wilcoxon rank-sum test for continuous variables. An ANOVA test was fitted to predict the variables associated with a highly protective antibody titre. All analyses were performed using SPSS version 19.0 (SPSS, Chicago, IL, USA).

## 3. Results

**Figure 1:**
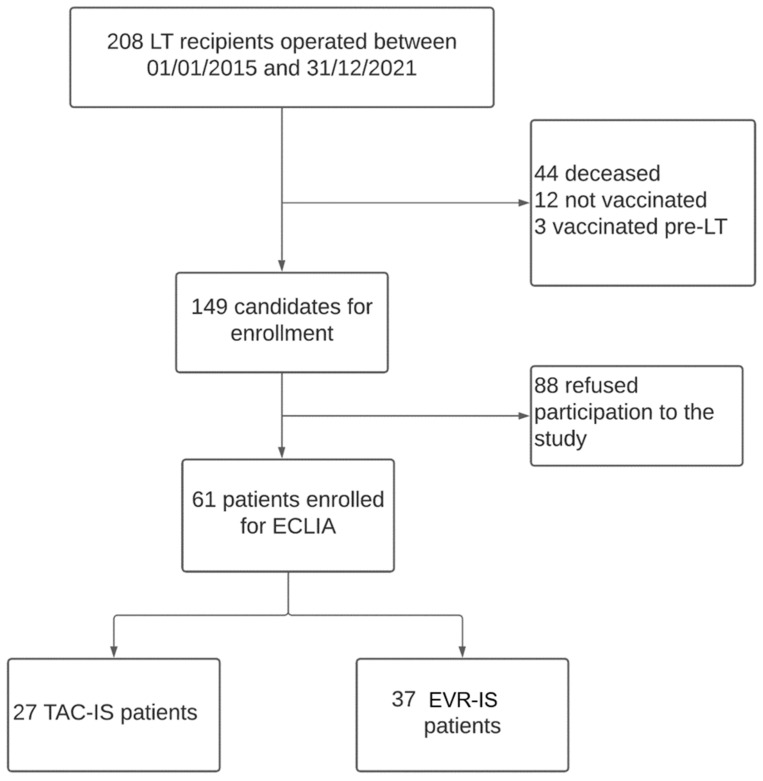
Study design flowchart.

Two hundred and eight patients underwent LT between January 2015 and December 2021 at our centre. One-hundred and forty-seven patients were excluded from the current analysis because they did not fulfil the inclusion criteria or they did not consent to participate in the study. The study cohort included 61 LT recipients (Figure 1). Twenty-four (40%) patients were undergoing TAC-IS, while 37 (60%) were undergoing EVR-IS.

### 3.1. Patient Demographics

The baseline characteristics of the two groups are summarised in Table 1. TAC-IS patients had a lower average BMI (*p* = 0.0275). However, the median age at transplant and the baseline liver and renal function were similar between the groups. In addition, both groups received vaccination after a median time of three years after LT (*p* = 0.28). In the TAC-IS group, twenty-three (96%) patients received the BNT162b2 vaccination and one (4%) received mRNA-1273, while, in the NO-TAC-IS group, 35 (95%) patients received BNT162b2 and two (5%) received heterologous vaccinations (two BNT162b2 doses and one mRNA-1273 dose). In the TAC-IS group, 9 patients (37%) were taking TAC monotherapy, while 15 (63%) took TAC in combination with EVR. Furthermore, in the EVR-IS group, 14 patients (38%) were taking EVR monotherapy, while 23 (62%) were taking TAC-EVR combination therapy.

### 3.2. HR

**Figure 2:**
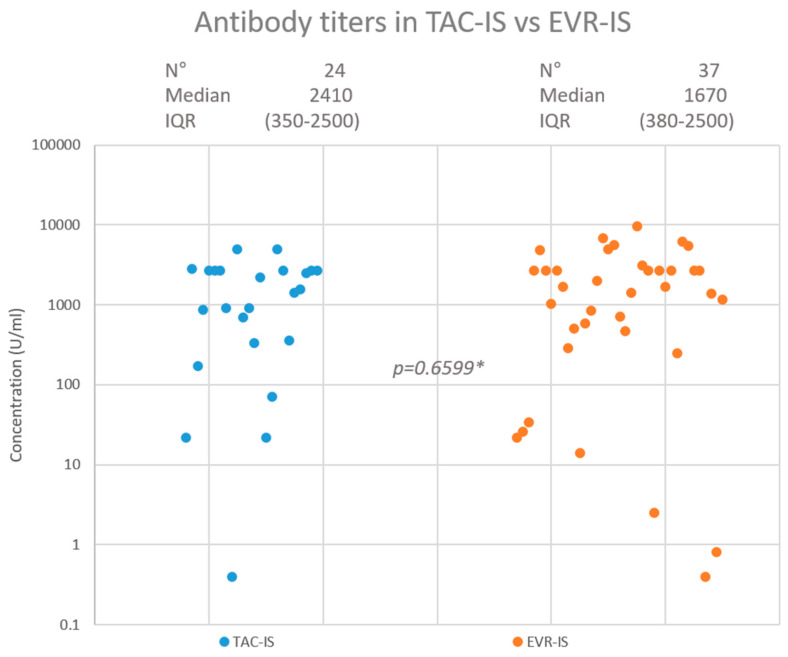
Neutralising antibody response in TAC-IS and EVR-IS patients. * Mann-Whitney test.

After a median of 116 (range = 89–154) days from the third vaccination dose, 23 (95.8%) patients had developed a positive antibody titre in the TAC-IS group compared to 35 (94.6%) in the EVR-IS group (*p* = 0.8269). A highly protective antibody titre was achieved in 20 (83.3%) and 30 (81.1%) patients in the TAC-IS and EVR-IS groups, respectively (*p* = 0.8231). The median antibody titres were 2410 (interquartile range: 350–2500) U/mL and 1670 (interquartile range: 380–2500) U/mL in the TAC-IS and EVR-IS groups, respectively (*p* = 0.9450). Figure 2 summarises the HR findings. In the EVR-IS group, 2 (5.4%) patients contracted COVID-19 during follow up, 1 of which was asymptomatic. The other had mild symptoms and did not require hospitalisation. No patient in the TAC-IS group was infected with COVID-19 during the study period.

### 3.3. Predictive Factors for a Highly Protective Antibody Titre

The 50 patients (from both groups) with highly protective antibody titres were compared with the 11 patients with a weakly protective or null HR (Table 2). Univariate analysis found that patients with a weak or null HR had a significantly higher creatininaemia (1.69 ± 1.01 vs. 1.16 ± 0.42 mg/dL; *p* = 0.0064) and lower eGFR (52.80 ± 28 vs. 68.23 ± 20.45 mL/min *p* = 0.0385). Significant differences were also found in total bilirubinaemia and blood γGT, although in both groups the average values were within the normal range.

## 4. Discussion

The present study compared the HR of LT recipients receiving TAC vs. EVR to three doses of vaccination against SARS-CoV-2 with BNT162b2 or mRNA-1273. This is the first study to investigate whether the type of tailored IS regimen can predict the HR to SARS-CoV-2 vaccination. Almost all patients achieved a protective HR, with no differences between patients who received TAC-IS or EVR-IS after LT. Furthermore, a highly protective antibody titre was also achieved in the majority of patients, with no difference between the groups.

The HR after SARS-CoV-2 vaccination is of great importance in the LT population because the disease, as in other clinically vulnerable populations, may lead to severe pneumonia and carry an increased risk of death. Several studies have shown that LT patients infected with SARS-CoV-2 often develop severe COVID-19 disease, leading to hospitalisation in 64–86% of cases; admission to the intensive care unit (ICU) in 10–31% of cases; and mortality in 12–20% of cases [4,5,15,16,17]. Given the intrinsic clinical vulnerability of LT patients and the uncertain effect of IS, obtaining a response to vaccination had been a key concern at the beginning of the vaccination campaign. Early reports on solid organ recipients have observed sub-optimal rates of positive response to the first vaccination cycle [18,19]. There were significant improvements following the second vaccination cycle, with 47.5–75% of LT recipients developing an HR, although titres were still markedly lower than those of the healthy control group [20,21,22]. The benefit of a third dose has been documented in numerous studies, both in solid organ transplantation in general and, specifically, in the LT population, where positive HR rates as high as 91–98% have been reported [23,24,25,26]. Our results (>90% positive antibody titres) are in line with these findings. These excellent results may be due to the specificity of LT recipients in the transplant panorama as they represent a privileged group due to the more often benign graft–host interaction and reduced (and sometimes even absent) need for IS [1,27,28]. In fact, studies on solid-organ recipients have found LT to be a predictive factor for a positive HR [29,30]. Most recently, in a study on both humoral and cellular responses to vaccination in liver and kidney recipients, Furian et al. [31] have confirmed that LT patients show a markedly increased humoral response against all SARS-CoV-2 spike epitopes. The authors also found that the vaccine elicited a humoral response recognising all strains of SARS-CoV-2, particularly the original Wuhan strain. Due to the magnitude of the threat that SARS-CoV-2 may pose to transplant patients, it is of great importance to identify the subset of patients at risk of an absent response. Similarly, the identification of a weak, rather than a highly protective response, could be critical. McMahan et al. [32] have identified anti-RBD titres of >100 U/mL as the threshold for a highly protective response in macaques. Other studies confirmed that as much as 3% of mean convalescent neutralising antibody concentrations offered protection from infection [33]. For example, Dimeglio et al. have found a concentration of 146 BAU/mL to be 90% protective in humans [34]. This was the threshold used in this study.

IS represents a major variable in LT patients which has not been specifically studied so far, although some correlations have been previously identified. For instance, Marion et al. [22] reported that patients taking the EVR–TAC combination had improved immune response compared to those taking tacrolimus and mycophenolate. Other studies have confirmed the negative effect of mycophenolate on HR in LT recipients [9,19,21,35]. In this study, we therefore excluded all patients taking mycophenolate and focused on the effect of IS based on TAC and EVR. We found that, in long-term LT recipients, HR is not influenced by whether they are taking Calcineurin inhibitors (CNIs) or mammalian-Target of Rapamycin inhibitors (mTORis) as IS nor the dose. Our data confirmed that both TAC and EVR blood levels were not different between highly protective responders and weak/null responders, suggesting that the choice of IS based on either TAC or EVR can be safely made according to the 2020 Italian Consensus Working Group guidelines [12] in relation to vaccination response. On the other hand, even though the paucity of patients allowed only univariate analysis, we identified poor renal function as the main determinant of the development of a highly protective HR. This finding has been already reported by other investigators, reflecting the importance of renal function in immunologic mechanisms and in LT patients [19,21,24]. Age was also previously identified as a factor influencing HR, but this was not observed in our cohort [19,20,21]. This may be due to the fact that our cohort was relatively young and numerically too small to detect subtle differences. Although there were two COVID-19 cases in the EVR-IS group and none in the TAC-IS group, the difference was not significant.

This is the first study addressing the role of tailored IS therapy, either TAC or EVR based on the HR after SARS-CoV-2 vaccination in LT recipients. The study was designed to include rigorous predefined thresholds for IS [12], the selection of only mRNA-based vaccines, and the use of a single immunoassay (ECLIA) for all patients. This is also the first study in which the role of monotherapy was investigated in regard to HR after vaccination. In fact, about 30% of the cohort were taking monotherapy (TAC or EVR) and both regimens seemed to not influence the HR or the antibody titres. This data must be confirmed in a larger specific study on the minimisation or withdrawal of IS [2,36,37,38]. Following the Italian Consensus Guidelines [12], in the present cohort, the choice of IS and their dose was made by clinicians based on the indication for LT and the specific disease aetiology or based on the expected IS side effects and patient comorbidities.

We acknowledge that this study carries some limitations. As in other similar studies, the number of participants was relatively low [23,24] and this did not allow multivariate analyses to be performed. The range of the time period between the 3rd dose and the ECLIA was also large, possibly influencing antibody titres: they may have been lower when measured early (at 30 days), having not reached a peak, or late (135 days), having already reached a plateau and started to decrease [21]. As the study included only patients taking TAC or EVR, it cannot be conclusive for patients receiving other IS regimens, such as antimetabolites and corticosteroids. Moreover, only HR, and not cellular immunity, was investigated. Most patients were on combination therapy and were assigned to the two groups based on the blood concentration. We also only analysed one time point and do not have data on changes in time; nonetheless, other studies have found that, while antibody titres seem to wean faster in LT patients, a third-dose booster delivers acceptable durability results [39,40,41,42].

## 5. Conclusions

In conclusion, a third dose of the SARS-CoV-2 vaccine yielded very high rates of a positive HR and a highly protective HR. The use of either TAC-IS or EVR-IS in long-term LT recipients did not significantly influence HR relative to the other. Lastly, renal disease appears to be a risk factor for a weak or null HR.

## Figures and Tables

**Table 1:** Patients’ baseline characteristics.

	TAC-IS (n = 24)	EVR-IS (n = 37)	*p*-Value
AGE (years)	58.5 (±8.8)	60.3 (±10.8)	0.50
SEX (%, male)	75% (18)	86% (32)	0.32
Time elapsed between LT and III DOSE (years)	3.4 (±2.2)	3.1 (±1.9)	0.28
MELD at LT	23.3 (±4)	22.7 (±5.6)	0.52
BMI (kg/m^2^)	25.4 (±3.4)	28.0 (±5.1)	**0.0275**
DIABETES (n, %)	6 (25%)	8 (22%)	0.77
TAC MONOTHERAPY (n, %)	9 (37%)	-	-
EVR MONOTHERAPY (n, %)	-	14 (38%)	-
TAC-EVRCOMBINATION THERAPY (n, %)	15 (63%)	23 (62%)	1
TAC trough blood concentration (ng/mL)	6.3 (±1.4)	3.6 (±1)	**0.0001**
EVR trough blood concentration (ng/mL)	1.9 (±1.1)	2.9 (±1.6)	**0.0159**
Hb (g/dL)	13.8 (±1.9)	13.5 (±1.8)	0.56
LEUKOCYTES (/μL)	6008 (±1765)	5858 (±2406)	0.79
PLT (×1000/μL)	185.4 (±50.5)	202.3 (±79.1)	0.36
SERUM IRON (μg/dL)	86.9 (±41.7)	63.8 (±33.4)	**0.0197**
AST (U/L)	33.3 (±16.9)	31.1 (±11.7)	0.56
ALT (U/L)	37.9 (±24.7)	30.4 (±17.2)	0.17
γGT (UI/L)	44.1 (±38.9)	51 (±51.1)	0.57
ALKALINE PHOSPHATASE (UI/L)	118.3 (±101)	106 (±44.7)	0.52
T-BILIRUBIN (mg/dL)	0.7 (±0.3)	0.8 (±0.4)	0.47
CREATININE (mg/dL)	1.1 (±0.3)	1.4 (±0.7)	0.06
eGFR (mL/min)	70.1 (±18.9)	62.5 (±24.4)	0.20

**Table 2:** Characteristics of patients with a highly protective vs. weak/null HR.

	Weak/Null HR (n = 11)	Highly Protective HR (n = 50)	*p*-Value
AGE (years)	61.2 (±10.2)	59.2 (±10)	0.55
SEX (Male)	82% (9)	82% (41)	1
Time elapsed between LT and III DOSE (years)	3.7 (±1.7)	3.2 (±2.1)	0.45
MELD	21.1 (±2.7)	23.3 (±5.3)	0.18
BMI (kg/m^2^)	27.5 (±3.2)	26.9 (±4.9)	0.70
DIABETES (%)	27% (3)	24% (12)	1
TAC trough blood concentration (ng/mL)	5.4 (±1.6)	4.9 (±1.9)	0.58
EVR trough blood concentration (ng/mL)	2.7 (±1.2)	2.6 (±1.5)	0.77
Hb (g/dL)	13.4 (±1.4)	13.6 (±2)	0.70
LEUKOCYTES (/μL)	5192 (±2393)	6077 (±2100)	0.22
PLT (×1000/μL)	188.3 (±74.6)	197.3 (±68.7)	0.70
SERUM IRON (μg/dL)	58.1 (±30.3)	76.0 (±39.4)	0,18
AST (U/L)	30.5 (±11.1)	32.3 (±14.5)	0.70
ALT (U/L)	28.4 (±12.9)	34.4 (±21.9)	0.38
γGT (UI/L)	74.8 (±73)	42.4 (±36.9)	**0.0351**
ALKALINE PHOSPHATASE (UI/L)	122.7 (±57)	108.2 (±74.9)	0.55
T-BILIRUBIN (mg/dL)	0.6 (±0.2)	0.8 (±0.1)	**0.0028**
CREATININE(mg/dL)	1.7 (±1)	1.2 (±0.4)	**0.0064**
eGFR (mL/min)	52.8 (±28.1)	68.2 (±20.5)	**0.0385**

## Data Availability

The data underlying this article cannot be shared publicly in order to maintain the privacy of individuals who participated in the study. However, data will be shared upon reasonable request to the corresponding author.

## References

[1] Toti L., Manzia T.M., Sensi B., Blasi F., Baiocchi L., Lenci I., Angelico R., Tisone G. (2021). Towards tolerance in liver transplantation. Best Pract. Res. Clin. Gastroenterol..

[2] Manzia T.M., Angelico R., Gazia C., Lenci I., Milana M., Ademoyero O.T., Pedini D., Toti L., Spada M., Tisone G. (2019). *De novo* malignancies after liver transplantation: The effect of immunosuppression-personal data and review of literature. World J. Gastroenterol..

[3] Busuttil R.W., Lake J.R. (2004). Role of tacrolimus in the evolution of liver transplantation. Transplantation.

[4] Belli L.S., Fondevila C., Cortesi P.A., Conti S., Karam V., Adam R., Coilly A., Ericzon B.G., Loinaz C., Cuervas-Mons V. (2021). Protective Role of Tacrolimus, Deleterious Role of Age and Comorbidities in Liver Transplant Recipients with COVID-19: Results from the ELITA/ELTR Multi-center European Study. Gastroenterology.

[5] Webb G.J., Marjot T., Cook J.A., Aloman C., Armstrong M.J., Brenner E.J., Catana M.A., Cargill T., Dhanasekaran R., García-Juárez I. (2020). Outcomes following SARS-CoV-2 infection in liver transplant recipients: An international registry study. Lancet Gastroenterol. Hepatol..

[6] Mauriello A., Scimeca M., Amelio I., Massoud R., Novelli A., Di Lorenzo F., Finocchiaro S., Cimino C., Telesca R., Chiocchi M. (2021). Thromboembolism after COVID-19 vaccine in patients with preexisting thrombocytopenia. Cell Death Dis..

[7] Oldani C., Vanni G., Buonomo O.C. (2020). COVID-19 Unintended Effects on Breast Cancer in Italy after the Great Lockdown. Front. Public Health.

[8] Amelio I., Bertolo R., Bove P., Buonomo O.C., Candi E., Chiocchi M., Cipriani C., Di Daniele N., Ganini C., Juhl H. (2020). Liquid biopsies and cancer omics. Cell Death Discov..

[9] Meunier L., Malezieux E., Ursic Bedoya J., Faure S., Echenne M., Debourdeau A., Meszaros M., Pageaux G.P. (2022). Mycophenolate mofetil discontinuation increases severe acute respiratory syndrome coronavirus 2 vaccine response in nonresponder liver transplantation recipients: A proof of concept. Liver Transpl..

[10] de Boer S.E., Berger S.P., van Leer-Buter C.C., Kroesen B.J., van Baarle D., Sanders J.F., OPTIMIZE Study Group (2022). Enhanced Humoral Immune Response after COVID-19 Vaccination in Elderly Kidney Transplant Recipients on Everolimus versus Mycophenolate Mofetil-containing Immunosuppressive Regimens. Transplantation.

[11] von Elm E., Altman D.G., Egger M., Pocock S.J., Gøtzsche P.C., Vandenbroucke J.P., STROBE Initiative (2008). The Strengthening the Reporting of Observational Studies in Epidemiology (STROBE) statement: Guidelines for reporting observational studies. J. Clin. Epidemiol..

[12] Cillo U., De Carlis L., Del Gaudio M., De Simone P., Fagiuoli S., Lupo F., Tisone G., Volpes R. (2020). Immunosuppressive regimens for adult liver transplant recipients in real-life practice: Consensus recommendations from an Italian Working Group. Hepatol. Int..

[13] Jia J.J., Lin B.Y., He J.J., Geng L., Kadel D., Wang L., Yu D.D., Shen T., Yang Z., Ye Y.F. (2014). “Minimizing tacrolimus” strategy and long-term survival after liver transplantation. World J. Gastroenterol..

[14] Jochum S., Kirste I., Hortsch S., Grunert V.P., Legault H., Eichenlaub U., Kashlan B., Pajon R. (2022). Clinical Utility of Elecsys Anti-SARS-CoV-2 S Assay in COVID-19 Vaccination: An Exploratory Analysis of the mRNA-1273 Phase 1 Trial. Front. Immunol..

[15] Colmenero J., Rodríguez-Perálvarez M., Salcedo M., Arias-Milla A., Muñoz-Serrano A., Graus J., Nuño J., Gastaca M., Bustamante-Schneider J., Cachero A. (2021). Epidemiological pattern, incidence, and outcomes of COVID-19 in liver transplant patients. J. Hepatol..

[16] Becchetti C., Zambelli M.F., Pasulo L., Donato M.F., Invernizzi F., Detry O., Dahlqvist G., Ciccarelli O., Morelli M.C., Fraga M. (2020). COVID-19 in an international European liver transplant recipient cohort. Gut.

[17] Dumortier J., Duvoux C., Roux O., Altieri M., Barraud H., Besch C., Caillard S., Coilly A., Conti F., Dharancy S. (2021). COVID-19 in liver transplant recipients: The French SOT COVID registry. Clin. Res. Hepatol. Gastroenterol..

[18] Marion O., Del Bello A., Abravanel F., Couat C., Faguer S., Esposito L., Hebral A.L., Izopet J., Kamar N. (2021). Safety and Immunogenicity of Anti-SARS-CoV-2 Messenger RNA Vaccines in Recipients of Solid Organ Transplants. Ann. Intern. Med..

[19] Rabinowich L., Grupper A., Baruch R., Ben-Yehoyada M., Halperin T., Turner D., Katchman E., Levi S., Houri I., Lubezky N. (2021). Low immunogenicity to SARS-CoV-2 vaccination among liver transplant recipients. J. Hepatol..

[20] Guarino M., Esposito I., Portella G., Cossiga V., Loperto I., Tortora R., Cennamo M., Capasso M., Terracciano D., Galeota Lanza A. (2022). Humoral Response to 2-dose BNT162b2 mRNA COVID-19 Vaccination in Liver Transplant Recipients. Clin. Gastroenterol. Hepatol..

[21] Raszeja-Wyszomirska J., Janik M.K., Wójcicki M., Milkiewicz P. (2022). SARS-CoV-2 vaccination in liver transplant recipients: Factors affecting immune response and refusal to vaccine. Pol. Arch. Intern. Med..

[22] Marion O., Del Bello A., Abravanel F., Faguer S., Esposito L., Laure Hebral A., Bellière J., Izopet J., Kamar N. (2021). Predictive Factors for Humoral Response after 2-dose SARS-CoV-2 Vaccine in Solid Organ Transplant Patients. Transplant. Direct.

[23] Davidov Y., Indenbaum V., Tsaraf K., Cohen-Ezra O., Likhter M., Ben Yakov G., Halperin R., Levy I., Mor O., Agmon-Levin N. (2022). A third dose of the BNT162b2 mRNA vaccine significantly improves immune responses among liver transplant recipients. J. Hepatol..

[24] Toniutto P., Cussigh A., Cmet S., Bitetto D., Fornasiere E., Fumolo E., Fabris M., D’Aurizio F., Fabris C., Grillone L. (2023). Immunogenicity and safety of a third dose of anti-SARS-CoV-2 BNT16b2 vaccine in liver transplant recipients. Liver Int..

[25] Kamar N., Abravanel F., Marion O., Esposito L., Hebral A.L., Médrano C., Guitard J., Lavayssière L., Cointault O., Nogier M.B. (2022). Anti SARS-CoV-2 spike protein and neutralizing antibodies at 1 and 3 months after three doses of SARS-CoV-2 vaccine in a large cohort of solid organ transplant patients. Am. J. Transplant..

[26] Kamar N., Abravanel F., Marion O., Couat C., Izopet J., Del Bello A. (2021). Three Doses of an mRNA COVID-19 Vaccine in Solid-Organ Transplant Recipients. N. Engl. J. Med..

[27] Angelico R., Sensi B., Manzia T.M., Tisone G., Grassi G., Signorello A., Milana M., Lenci I., Baiocchi L. (2021). Chronic rejection after liver transplantation: Opening the Pandora’s box. World J. Gastroenterol..

[28] Piazza A., Adorno D., Poggi E., Borrelli L., Buonomo O., Pisani F., Valeri M., Torlone N., Camplone C., Monaco P.I. (1998). Flow cytometry crossmatch: A sensitive technique for assessment of acute rejection in renal transplantation. Transplant. Proc..

[29] Strauss A.T., Hallett A.M., Boyarsky B.J., Ou M.T., Werbel W.A., Avery R.K., Tobian A.A.R., Massie A.B., Hamilton J.P.A., Garonzik-Wang J.M. (2021). Antibody Response to Severe Acute Respiratory Syndrome-Coronavirus-2 Messenger RNA Vaccines in Liver Transplant Recipients. Liver Transpl..

[30] Tauzin A., Beaudoin-Bussières G., Gong S.Y., Chatterjee D., Gendron-Lepage G., Bourassa C., Goyette G., Racine N., Khrifi Z., Turgeon J. (2022). Humoral immune responses against SARS-CoV-2 Spike variants after mRNA vaccination in solid organ transplant recipients. iScience.

[31] Furian L., Russo F.P., Zaza G., Burra P., Hartzell S., Bizzaro D., Di Bello M., Di Bella C., Nuzzolese E., Agnolon C. (2022). Differences in Humoral and Cellular Vaccine Responses to SARS-CoV-2 in Kidney and Liver Transplant Recipients. Front. Immunol..

[32] McMahan K., Yu J., Mercado N.B., Loos C., Tostanoski L.H., Chandrashekar A., Liu J., Peter L., Atyeo C., Zhu A. (2021). Correlates of protection against SARS-CoV-2 in rhesus macaques. Nature.

[33] Dimeglio C., Herin F., Martin-Blondel G., Miedougé M., Izopet J. (2022). Antibody titers and protection against a SARS-CoV-2 infection. J. Infect..

[34] Khoury D.S., Cromer D., Reynaldi A., Schlub T.E., Wheatley A.K., Juno J.A., Subbarao K., Kent S.J., Triccas J.A., Davenport M.P. (2021). Neutralizing antibody levels are highly predictive of immune protection from symptomatic SARS-CoV-2 infection. Nat. Med..

[35] Grupper A., Katchman H. (2022). SARS-CoV-2 Vaccines: Safety and Immunogenicity in Solid Organ Transplant Recipients and Strategies for Improving Vaccine Responses. Curr. Transplant. Rep..

[36] Manzia T.M., Angelico R., Baiocchi L., Toti L., Ciano P., Palmieri G., Angelico M., Orlando G., Tisone G. (2013). The Tor Vergata weaning of immunosuppression protocols in stable hepatitis C virus liver transplant patients: The 10-year follow-up. Transpl. Int..

[37] Hirschfeld C.B., Shaw L.J., Williams M.C., Lahey R., Villines T.C., Dorbala S., Choi A.D., Shah N.R., Bluemke D.A., Berman D.S. (2021). Impact of COVID-19 on Cardiovascular Testing in the United States versus the Rest of the World. JACC Cardiovasc. Imaging.

[38] COVIDSurg Collaborative, GlobalSurg Collaborative (2022). SARS-CoV-2 infection and venous thromboembolism after surgery: An international prospective cohort study. Anaesthesia.

[39] Passenberg M., Authorsen-Grudmann R., Frey A., Korth J., Zmudzinski J., Anastasiou O.E., Möhlendick B., Schmidt H., Rashidi-Alavijeh J., Willuweit K. (2023). Durability of Immune Response after Application of a Third Dose of SARS-CoV-2 Vaccination in Liver Transplant Recipients. Vaccines.

[40] Willuweit K., Frey A., Passenberg M., Korth J., Saka N., Anastasiou O.E., Möhlendick B., Schütte A., Schmidt H., Rashidi-Alavijeh J. (2022). Patients with Liver Cirrhosis Show High Immunogenicity upon COVID-19 Vaccination but Develop Premature Deterioration of Antibody Titers. Vaccines.

[41] Timmermann L., Globke B., Lurje G., Schmelzle M., Schöning W., Öllinger R., Pratschke J., Eberspächer B., Drosten C., Hofmann J. (2021). Humoral Immune Response following SARS-CoV-2 Vaccination in Liver Transplant Recipients. Vaccines.

[42] Luxenburger H., Reeg D.B., Lang-Meli J., Reinscheid M., Eisner M., Bettinger D., Oberhardt V., Salimi Alizei E., Wild K., Graeser A. (2023). Boosting compromised SARS-CoV-2-specific immunity with mRNA vaccination in liver transplant recipients. J. Hepatol..

